# Multifaceted roles of *FD* gene family in flowering, plant architecture, and adaptation

**DOI:** 10.3389/fpls.2025.1602756

**Published:** 2025-06-20

**Authors:** Hui Yang, Meiling Zhong, Yucheng Liu, Liyu Chen, Baohui Liu, Fanjiang Kong, Hong Li, Lin Yue

**Affiliations:** Guangdong Provincial Key Laboratory of Plant Adaptation and Molecular Design, Guangzhou Key Laboratory of Crop Gene Editing, Innovative Center of Molecular Genetics and Evolution, School of Life Sciences, Guangzhou University, Guangzhou, China

**Keywords:** *FD*, floral transition, plant development, adaptation, crop breeding

## Abstract

*FD* gene family encodes transcription factors with a basic region/leucine zipper (bZIP) domain that play an essential role in floral transition regulation, which is vital for plants’ reproduction. Recent studies have uncovered additional functions for *FD* gene family in plant development, hormone signaling, and response to environmental cues. These pleiotropic roles make them promising targets for modern crops’ breeding. Here, we systematically review the diverse functions and regulation mechanisms of *FD* gene family in model plants and several crops, to provide important insights into their roles. By summarizing the current understanding of their molecular mechanisms, we aim to highlight their potential as key targets for improving crop yield, stress tolerance, and adaptation to changing climates. Furthermore, we propose future research directions, these efforts will pave the way for the effective utilization of them in modern crop breeding programs.

## 1 Introduction

Plant basic region/leucine zipper (bZIP) transcription factors function in many biological processes (Corrêa et al., 2008; Dröge-Laser et al., 2018; Yue et al., 2023). In the model species *Arabidopsis thaliana*, bZIP genes are classified into 13 groups (designated A-M), most of which display group-specific properties. FD and its homolog *FD PARALOG* (*FDP*) belong to group A and are involved in the control of floral transition, which is an important developmental process for angiosperms (Dröge-Laser et al., 2018; Romera-Branchat et al., 2020). Appropriate flowering time ensures reproduction success, seed set, and crop yield. Flowering time is regulated by signals from different pathways, such as age pathway, autonomous pathway, gibberellin pathway, photoperiod pathway, and vernalization pathway (Fornara et al., 2010; Wang et al., 2024). To govern flowering time by integrating signals from multiple pathways, FLOWERING LOCUS T (FT), SUPPRESSOR OF OVEREXPRESSION OF CONSTANS1 (SOC1/AGL20), and LEAFY (LFY) are key floral integrators in promoting floral transition (Araki, 2001; Parcy, 2005; Lee and Lee, 2010; Hiraoka et al., 2013). FD, which is required for FT protein activity, also integrates flowering signals from different regulatory pathways (Abe et al., 2005; Wang et al., 2009; Benlloch et al., 2011; Seedat et al., 2013; Park et al., 2023). The FT protein is transported from the leaves to the SAM via the vascular tissue and interacts with FD to form the FT-FD complex, which in turn activates the expression of *SOC1*, *APETALA1* (*AP1*), and *LFY*, thereby promoting plant flowering (Abe et al., 2005; Wigge et al., 2005; Park et al., 2023). Recent studies report that these floral integrators influence agronomic traits at the same time (Cai et al., 2020; Han et al., 2021; Kou et al., 2022). Therefore, understanding the mechanisms underlying flowering regulation by FD has significant implications for plant breeding and crop productivity.

The architecture of plants is tightly controlled by the identity and activity of meristems: during floral induction, the SAM transforms from a vegetative meristem to an inflorescence meristem (Zhu et al., 2021). The florigen FT promotes floral transition, whereas its homologous protein TERMINAL FLOWER 1 (TFL1) from the same family functions oppositely (Liu et al., 2021). FD interacts with either FT or TFL1, and as a weak activator, FD is converted into a strong activator by FT but into a repressor by TFL1 (Ahn et al., 2006). Recent studies have uncovered roles for *FD* gene family in inflorescence structure, stem growth, bud formation, and flower development (Tsuji et al., 2013; Sussmilch et al., 2015; Dutta et al., 2021). The morphogenetic effects induced by *FD* has a strong impact on plant architecture, thus *FD* homologs play crucial roles in biomass accumulation and plant production.

Crop yield is reduced when plants are exposed to extreme environmental conditions such as high salt, drought, cold, and heat. Plant bZIP transcription factors are considered as abiotic stress regulators, such as AtbZIP15 and AtbZIP35-AtbZIP38, which are involved in abscisic acid (ABA) and stress signaling (Choi et al., 2000; Uno et al., 2000). Similar functions have been reported in *FD* homologs, which provides a valuable basis for crop yield study in the future.

In this review, we analyze the phylogenetics and protein structures of members of the FD family, explore recent advances of the novel roles of *FD* in various species, comprehensively reveal the regulatory mechanisms of *FD* in floral transition, plant development, and environmental signal response. Ultimately, we provide perspectives for their further utilization in crop breeding.

## 2 Divergence of *FD* homologs

Full-length amino acid sequences of *FD* homologs were obtained from the *Arabidopsis* database TAIR, Phytozome database, and NCBI database. AtbZIP68 and AtbZIP16 (belong to group G) were used as outgroup proteins (Dröge-Laser et al., 2018) (**Supplementary Table S1**). We performed multiple sequence comparisons using MEGA7 software and conducted a phylogenetic analysis using the maximum likelihood method (Kumar et al., 2016). NCBI Batch CD-Search website (https://www.ncbi.nlm.nih.gov/Structure/bwrpsb/bwrpsb.cgi) was used for structural domain prediction and MEME Motif Discovery (https://meme-suite.org/meme/tools/meme) was used to analyze the protein domains (Bailey et al., 2009). TBtools was used to visualize the results (Chen et al., 2020a).

**Figure 1:**
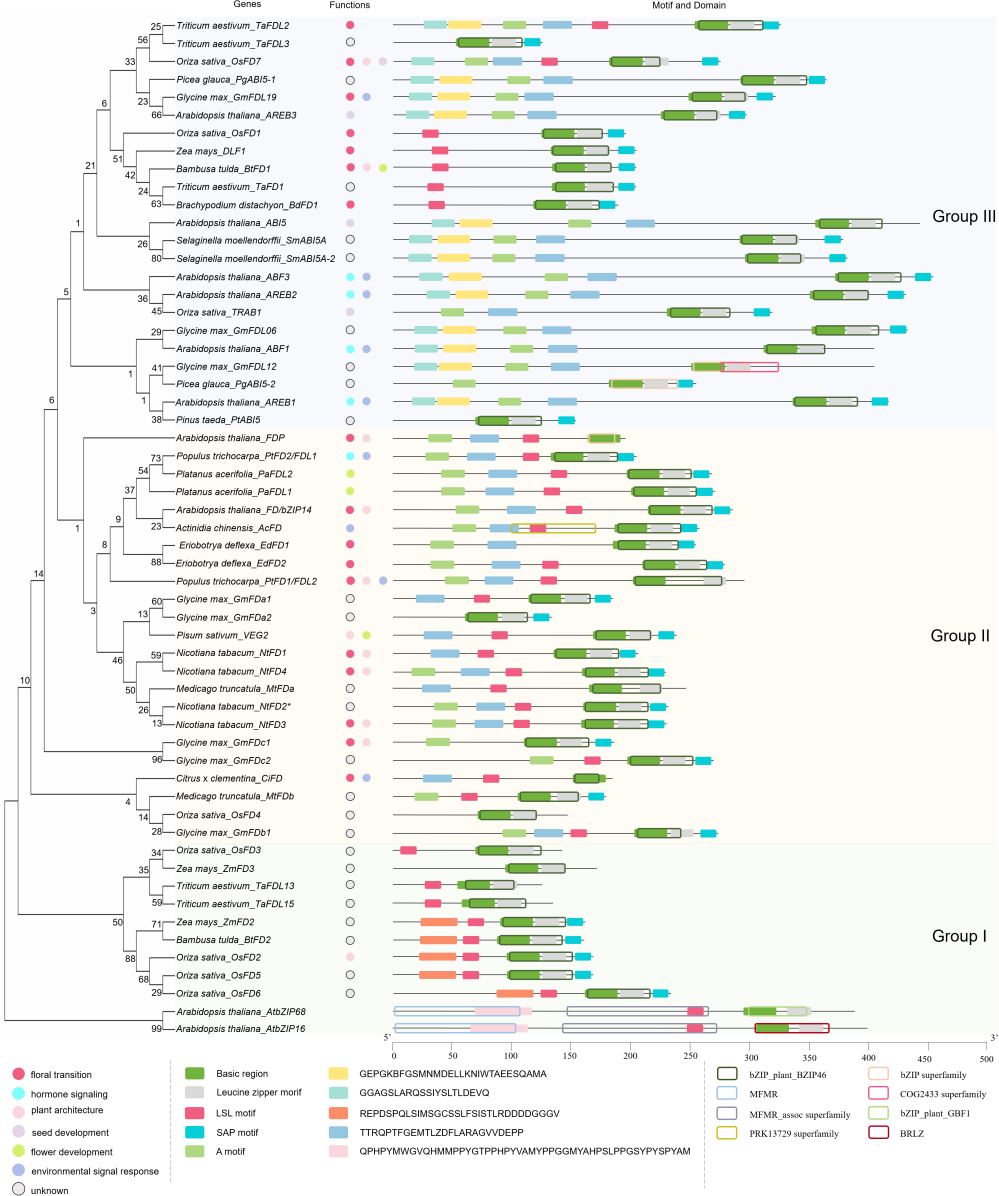
Phylogenetic analysis, protein structures, and functions of the FD proteins in plants. Basic region: DRRQKRMIKNRESAARSRARKQAYTNELE, leucine zipper motif: EVARLKEENARLKKQQEZLKE, SAP motif: LPKKKTLRRTSTAPF, A motif: TLPRTLSQKTVEEVWKDINLA, LSL motif: PPPATALSLNSGPGF.

Based on the analysis, the FD homologs could be divided into three groups (Group I-III) (**Figure 1**). All FD homologs contain a bZIP domain; the basic region is responsible for binding to specific DNA sequences, and the leucine zipper motif is required for dimerization (Dröge-Laser et al., 2018). The SAP (C-terminal phosphorylation) motif, which is conserved in most FD-like proteins, is important for FD phosphorylation and dimeric 14-3–3 protein bridge binding (Tsuji et al., 2013). All FD proteins in Group I are derived from monocots and lack the A motif. It’s reported that OsFD2 regulates inflorescence architecture (Tsuji et al., 2013), but the exact functions of most members in Group I are unresolved. All FD proteins in Group III are clustered together with ABI5, AREBs, and ABFs, most of them lack the LSL motif. They might have similar functions with ABI5, AREBs, and ABFs. Although the SAP, A, and LSL motifs are conserved in most Group II FD homologs, the FD proteins from various eudicots species have divergent functions.

## 3 *FD* gene family acts as a floral activator of the photoperiodic pathway

### 3.1 *FD* promotes flowering through florigen activation complex in *Arabidopsis*

The *Arabidopsis FD* gene encodes a bZIP protein of 285 amino acid residues, which is identified as *AtbZIP14* (At4g35900). *Arabidopsis FD* mRNA is distributed in the shoot apex and leaves with consistent 24-h (hour) rhythms, and its expression is significantly upregulated after seedling emergence (Abe et al., 2005; Park et al., 2023). The results of functional studies on loss and gain of function mutants suggest that *FD* regulates floral transition (Abe et al., 2005). *Arabidopsis fd-2* mutant has a late flowering phenotype (Wigge et al., 2005). Overexpression of *FD* results in early flowering, *35S:FD* enhances *35S:FT* phenotype; therefore, the amount of *FD* activity is one of the limiting factors for *35S:FT* plants (Abe et al., 2005).

**Figure 2:**
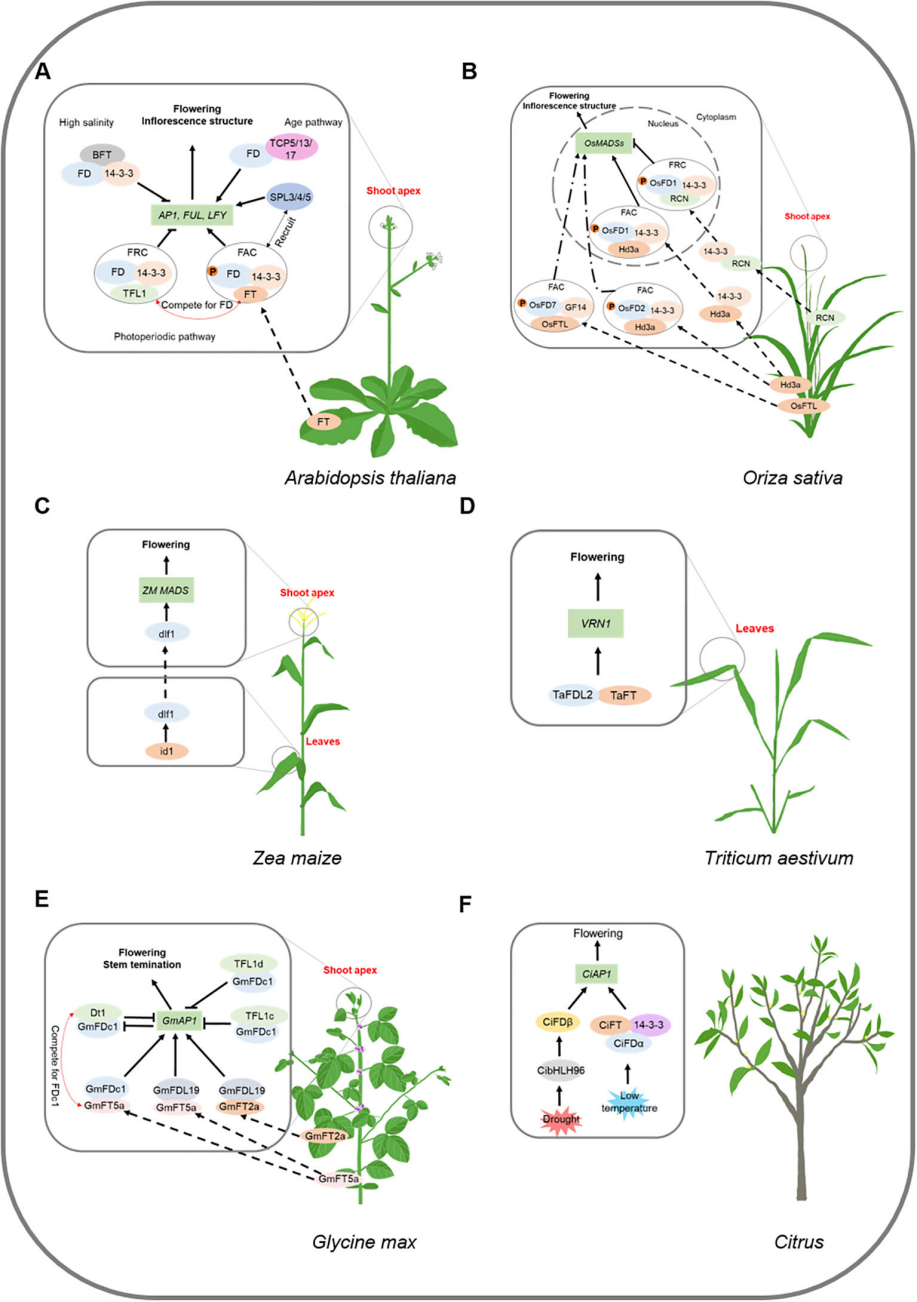
Regulation mechanisms of *FD* gene family in flowering pathways. Block letters represent proteins, italicized letters represent genes. Arrows represent facilitation and flathead arrows represent inhibition, dashed arrows represent translocation, dotted dashed arrows represent translocation and facilitation. P represents phosphorylation. IM represents inflorescence meristem, SAM represents shoot apical meristem, FM represents floral meristem, I_1_M represents primary inflorescence meristem, and I_2_M represents secondary inflorescence meristem. **(A)** *Arabidopsis* FT protein transports from leaves to shoot apex and work together with FD to promote floral transition, by activating the expression of *AP1*, *FUL*, *LFY*. FD is a floral integration factor that links photoperiod, age, and vernalization pathways. Under high salinity, BFT delays flowering, BFT protein competes with FT for FD binding. **(B)** At vegetative phase, FRC is formed. After Hd3a or OsFTL transports to the SAM, they compete with RCN for FAC formation. When the balance is shifted to the FAC, the reproductive program starts. **(C)** *dlf1* mediates floral inductive signals transmitting from leaves to the shoot apex, activated by *indeterminate1* (*id1*) in leaves. The targets of *dlf1* may be maize MADS-box homologs (ZM MADS). **(D)** TaFDL2 interacts with TaFT and binds to the promoter of *VRN1* to promote flowering. **(E)** Dt1 interacts with GmFDc1 and represses *GmAP1a* to repress flowering and stem termination. GmFT5a interferes with Dt1 for GmFDc1 binding and enhances the positive effect of GmFDc1 on *GmAP1* expression. GmFDL19 interacting with GmFT2a and GmFT5a to promote flowering. TFL1c and TFL1d interact with GmFDc1 and binds to the promoter of *GmAP1a* to repress its activity. **(F)** CiFD forms two different proteins (CiFDα and CiFDβ) by low temperature and drought, respectively. Under low temperature, CiFDα can interact with CiFT and Ci14-3–3 to promote the expression of CiAP1. Under drought conditions, CibHLH96 activates the expression of CiFD and forms more CiFDβ. CiFDβ can bind directly to the CiAP1 promoter independently of CiFT and Ci14-3-3.

In *Arabidopsis*, 14-3–3 proteins interact with FD and FT to form the ‘florigen activation complex’ (FAC) complex (Ho and Weigel, 2014). 14-3–3 proteins act as adaptor proteins to recognize and interact with the phosphorylated FD. FAC formation is dependent on the phosphorylation at position 282 (T282) of FD. That is, only after FD has been phosphorylated can active FAC be formed to induce flowering (Collani et al., 2019) (**Figure 2A**). Kawamoto et al. (2015) found that the calcium-dependent protein kinases (CDPKs) CPK4, CPK6, and CPK33 are good candidates for FD kinases. FD binds DNA (a strong preference for binding to G-box motifs) but does not activate transcription. FT acts as a transcriptional coactivator, increasing the enrichment of FD on floral time and floral homeotic genes such as *AP1*, *LFY*, *SOC1*, *FRUITFULL* (*FUL*), *SEPALLATA1* (*SEP1*), *SEP2*, and *SEP3* (Wigge et al., 2005; Ahn et al., 2006; Collani et al., 2019). *AP1* is a class A gene, *SEP1*, *SEP2*, and *SEP3* are class E genes during flower development (Krizek and Fletcher, 2005). TWIN SISTER OF FT (TSF), a paralog of FT, promotes flowering by enhancing the binding of FD to DNA (Collani et al., 2019).

### 3.2 FD-like proteins in rice act differentially in FAC formation

In rice, FD homologs (OsFD1-OsFD7) share a conserved bZIP and SAP motif (Tsuji et al., 2013; Kaur et al., 2021). OsFD1-Hd3a-14-3–3 complex activates *OsMADS15* (a homolog of *AP1*) and leads to early flowering (Tsuji et al., 2013). Silencing of OsFD7 correlates with late flowering and downregulation of MADS-box genes (e.g. *OsMADS14*, *OsMADS15*, and *OsMADS18*) involved in floral meristem development (Kaur et al., 2021) (**Figure 2B**). However, the regulation mechanism in rice is different from *Arabidopsis*. OsFD1 is located in the nucleus of shoot apex cells. The rice FT homolog, HEADING DATE 3a (Hd3a), translocates from the leaves to the shoot apex and binds 14-3–3 proteins in the cytoplasm. Then, the Hd3a-14-3–3 complex enters the nucleus and forms an FAC with OsFD1. The phosphorylated serine residue S192 in the OsFD1 SAP motif is recognized by 14-3–3 to facilitate the association between OsFD1 and Hd3a. FAC activates the transcription of *OsMADS15*, leading to floral induction (Taoka et al., 2011) (**Figure 2B**). Similar to OsFD1, OsFD7 is located in the nucleus of shoot apex cells. The rice FT homolog, OsFTL, translocates from the leaves to the shoot apex and binds OsGF14 proteins in the cytoplasm. Then, the OsFTL-OsGF14 complex enters the nucleus and forms an FAC with OsFD7. OsFD7 is phosphorylated by OsCDPK41 and OsCDPK49, and the interaction between OsGF14b and OsFD7 is dependent on this phosphorylation. FAC (OsFTL-OsGF14-OsFD7) activates the transcription of some floral meristem identity genes, leading to floral transition (Kaur et al., 2021) (**Figure 2B**). Unlike OsFD1 and OsFD7, OsFD2 shuttles between the cytoplasm and the nucleus. Normally, OsFD2 is restricted to the cytoplasm of shoot apex cells via its interaction with cytoplasmic 14-3–3 proteins. When Hd3a moves from the leaves to the shoot apex, the interaction between Hd3a and 14-3–3 initiates its nuclear translocation. The putative phosphorylation site, S164, within the SAP motif of OsFD2 is critical for the interaction between OsFD2 and 14-3-3 (**Figure 2B**). These results indicate that the FD function diverges among OsFD1, OsFD2, and OsFD7, but the formation of an FAC is essential for its function (Tsuji et al., 2013).

### 3.3 FD-like proteins have conserved functions in some important crops

Similar to *Arabidopsis* and rice, FD-like proteins in monocotyledonous plants such as maize, wheat, and bamboo also promote flowering (**Table 1**). Maize *delayed flowering1* (*dlf1*) mediates floral inductive signals transmitted from the leaves to the shoot apex, activated by *indeterminate1* (*id1*) in the leaves (Kozaki et al., 2004). The targets of *dlf1* might be the maize MADS-box homologs (*ZM MADS*) (Muszynski et al., 2006) (**Figure 2C**). The *id1* defined floral induction pathway may be unique to monocots, because no clear *id1* orthologs exist in the *Arabidopsis* genome (Colasanti et al., 2006). In wheat, the regulatory mechanism of TaFDL2 is similar to that in *Arabidopsis* FD. TaFDL2 interacts with TaFT and binds to the ACGT elements in the promoter of *VRN1* (homolog of *Arabidopsis AP1*) (**Figure 2D**). TaFT does not directly interact with the *VRN1* promoter but interacts with TaFDL2 proteins (Li and Dubcovsky, 2008). Overexpressing bamboo *BtFD1* in *Arabidopsis* leads to early flowering. BtFD1 binds to the ACGT motif of *AtAP1’s* promoter and upregulates the expression of *AtAP1* (Dutta et al., 2021). In barley (*Hordeum vulgare*), HvFDL4 and HvFDL5 have been shown to physically interact with 14-3–3 proteins in a phosphorylation-dependent manner. Serine-to-alanine substitutions at critical residues (S333A in HvFDL4 or S216A in HvFDL5) abolish their binding to 14-3–3 proteins, suggesting that phosphorylation at these sites is essential for complex formation (Li et al., 2015). However, the biological functions of these interactions remain uncharacterized.

**Table 1:** Functions of well-studied *FD* homologs in plant.

Species		Gene Name	Locus ID/Accession No.	Function	References
Arabidopsis	*Arabidopsis thaliana*	*FD*	At4g35900	floral transition, plant architecture	Abe et al., 2005
		*FDP*	At2g17770	floral transition, plant architecture	
Pea	*Pisum sativum*	*VEG2*	KP739949	plant architecture, flower development	Sussmilch et al., 2015
Alfalfa	*Medicago truncatula*	*MtFDa*	Medtr5g022780	floral transition, plant architecture	Cheng et al., 2021
Soybean	*Glycine max*	*GmFDc1*	Glyma.04G022100	floral transition, plant architecture	Sussmilch et al., 2015; Yue et al., 2021
		*GmFDL19*	Glyma.19G122800	floral transition, environmental signal response	Nan et al., 2014; Li et al., 2017; Takeshima et al., 2019
Poplar	*Populus trichocarpa*	*PtFD1/FDL2*	Potri.005G243400	floral transition, plant architecture, environmental signal response	Parmentier-Line and Coleman, 2015; Tylewicz et al., 2015
		*PtFD2/FDL1*	Potri.002G018400	hormone signaling, environmental signal response	
London plane	*Platanus acerifolia*	*PaFDL1*	MH845055	flower development	Cai et al., 2021
		*PaFDL2*	MH845056		
Tobacco	*Nicotiana tabacum*	*NtFD1*	KY306459	floral transition, plant architecture	Beinecke et al., 2018
		*NtFD3*	KY306461		
		*NtFD4*	KY306462		
Kiwifruit	*Actinidia chinensis*	*AcFD*	JX417425	environmental signal response	Varkonyi-Gasic et al., 2013
Wild loquat	*Eriobotrya deflexa*	*EdFD1*	KU319434	floral transition	Zhang et al., 2016
		*EdFD2*	KU319435		
Citrus	*Citrus* x *clementina*	*CiFD*	Ciclev10003845m	floral transition, environmental signal response	Ye et al., 2023
Rice	*Oriza sativa*	*OsFD1*	Os09g0540800	floral transition	Tsuji et al., 2013
		*OsFD2*	Os06g0720900	plant architecture	
		*OsFD7*	LOC_Os07g48660	floral transition, plant architecture, seed development	Kaur et al., 2021
Wheat	*Triticum aestivum*	*TaFDL2*	ABZ91908	floral transition	Li and Dubcovsky, 2008
Maize	*Zea mays*	*dlf1*	GRMZM2G067921	floral transition	Muszynski et al., 2006
	*Brachypodium distachyon*	*BdFD1*	Bradi4g36587	floral transition	Qin et al., 2019
Bamboo	*Bambusa tulda*	*BtFD1*	MF983712	floral transition, plant architecture, flower development	Dutta et al., 2021

The FD homologs in dicotyledons also have consistent functions (**Table 1**). Overexpression of soybean *GmFDc1* leads to early flowering, suggesting that *GmFDc1* activates the floral transition (Yue et al., 2021). GmFT5a binds to GmFDc1 and enhances the positive effect of GmFDc1 on *GmAP1* expression (Yue et al., 2021) (**Figure 2E**). Soybean *GmFDL19*-OE lines flower earlier than wild-type, which may be mediated by the direct up-regulation of *GmAP1a.* GmFDL19 also interacts with GmFT2a and GmFT5a to regulate flowering (Takeshima et al., 2019) (**Figure 2E**). GmFDL15 interacts with GmFT5b to promote flowering (Su et al., 2023). In poplar (*Populus trichocarpa*), the ectopic expression of *PtFD1* (*FDL2*) results in early flowering (Tylewicz et al., 2015). Tobacco *FD* homologs participate in flowering regulation, *NtFD1*, *NtFD3*, and *NtFD4* overexpression lines flower earlier than the wild-type (WT) (Beinecke et al., 2018). Overexpressing *EdFD1* or *EdFD2* in *Arabidopsis* results in early flowering. EdFT interacts with both EdFD1 and EdFD2 and regulates wild loquat flowering (Zhang et al., 2016).

## 4 FD-like proteins integrate endogenous and environmental stimuli

### 4.1 FD links the photoperiod, age, and vernalization pathways

SQUAMOSA PROMOTER BINDING LIKE (SPL) 3/4/5 are involved in the age pathway in *Arabidopsis* (Wang et al., 2009). SPL3/4/5 bind directly to the promoters of *AP1*, *LFY*, and *FUL* and recruit FD to these loci, mediating their activation by the FT-FD complex (Benlloch et al., 2011) (**Figure 2A**). SPL3/4/5 synergistically interact with the FT-FD module to induce flowering, linking the age and photoperiod pathways of flowering regulation (Jung et al., 2016). TCP5/13/17 (class II CIN TCP TFs) belonging to the Teosinte branched1/Cincinnata/proliferating cell factor (TCP) family act directly on the Telobox Motif cis-elements (GGACCA) of the *AP1* promoter by interacting with FD (**Figure 2A**) (Li et al., 2019). They act synergistically and additively with the FT-FD module to positively regulate the initiation of flowering in *Arabidopsis* (Li et al., 2019). Chromatin remodeler *HISTONE DEACETYLASE 19* (*HDA19*) preserves the identity of the inflorescence meristem (IM) in an age-dependent manner; in older *hda19* inflorescence apices, floral organ identity genes are abnormally expressed, and the mutation of *fd* enhances the timing of these reproductive defects in *hda19* (Gorham et al., 2018). FLOWERING H (FLH) is involved in the vernalization pathway of flowering. The early flowering Cape Verde Islands (CVI) allele of FLH requires the floral integrator FD to accelerate flowering (Seedat et al., 2013). These results confirm that FD is a central regulator of floral transition in the shoot meristem, which integrates signals from multiple pathways.

### 4.2 FD homologs integrate environment cues with flowering regulation

In citrus, *CiFD* forms two different proteins (CiFDα and CiFDβ) by alternatively splicing. Overexpressing CiFDα or CiFDβ in tobacco and trifoliate orange leads to early flowering (Ye et al., 2023). CiFDα and CiFDβ are induced by low temperatures and drought, respectively. Under low temperatures, CiFDα can interact with CiFT and Ci14-3–3 to form an FAC complex, which binds to the C-box element on the promoter of the floral meristem organization gene *CiAP1*, promoting its expression. In contrast, CiFDβ can bind directly to the *CiAP1* promoter independently of CiFT and Ci14-3-3. The transcription factor CibHLH96 binds to the E-box of the *CiFD* promoter to promote *CiFD* expression. CibHLH96 is induced by drought but not at low temperatures. Under drought conditions, CibHLH96 activates the expression of CiFD and forms more CiFDβ (**Figure 2F**) (Ye et al., 2023). These results show that both CiFDα and CiFDβ are involved in the regulation of citrus flowering, but they have different mechanisms (Ye et al., 2023). In Kiwifruit, *AcFD* regulates cold signal response. It is downregulated in dormant buds in response to cold treatment (Varkonyi-Gasic et al., 2013). A member of soybean bZIP family group A, *GmFDL19*, is involved in abiotic stress tolerance and floral transition (Nan et al., 2014; Li et al., 2017; Yue et al., 2023). The tolerance to drought and salt stress is enhanced in *GmFDL19*-OE lines by upregulating ABA/stress-responsive genes and reducing the accumulation of Na^+^ ion content, and ectopic expression of *GmFDL19* in soybean causes early flowering (Nan et al., 2014; Li et al., 2017).

## 5 *FD* gene family regulates plant morphogenesis

### 5.1 Inflorescence meristem identity and floral organ development

**Figure 3:**
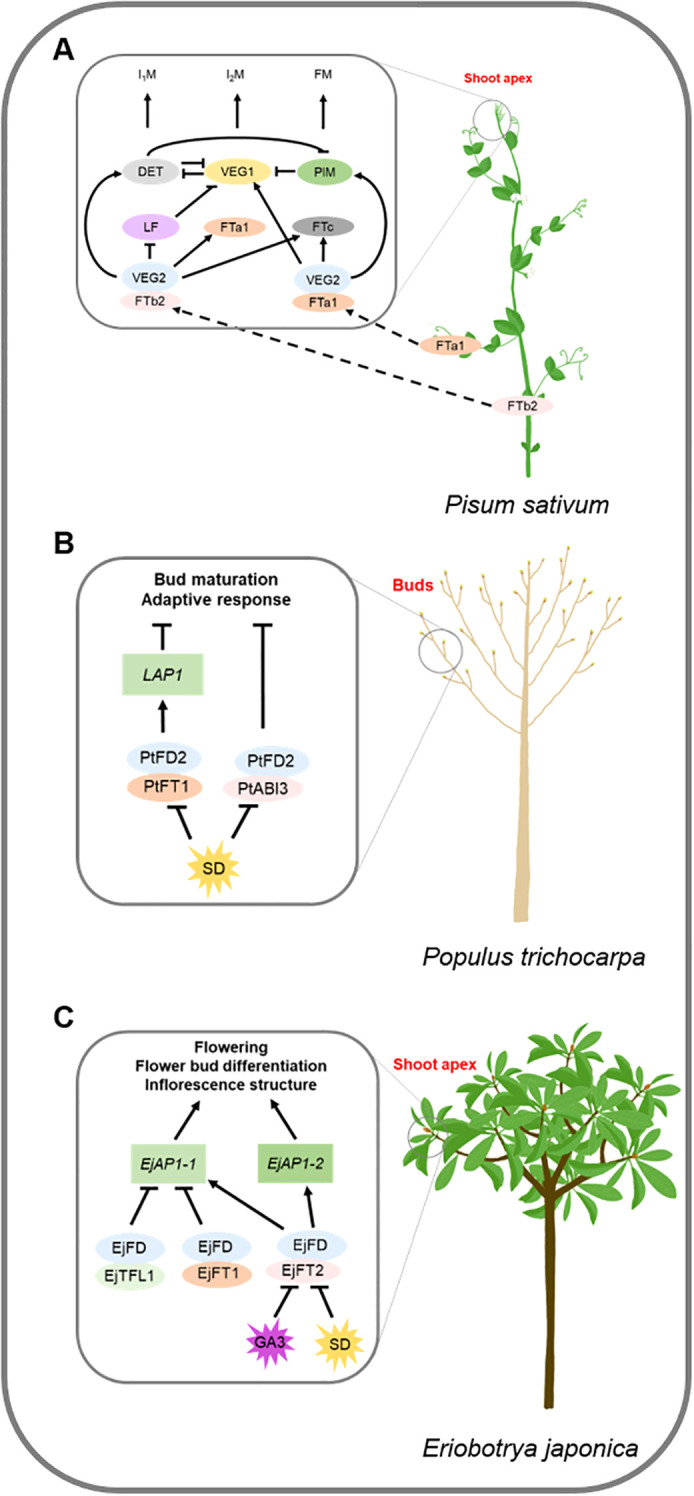
Regulation mechanisms of *FD* gene family in environment signaling and plant development pathways. Block letters represent proteins, italicized letters represent genes. Arrows represent facilitation and flathead arrows represent inhibition, dashed arrows represent translocation. **(A)** VEG2 interacts with FTb2 in shoot apex to promote I_1_M identity through *DET*, *LF*, *FTa1*, and *FTc*. VEG2 interacts with FTa1 in shoot apex to promote I_2_M identity through *VEG1* and *FTc*. VEG2 also involves in the regulation of floral architecture through regulating *PIM*. **(B)** PtFT- PtFD2 (FDL1) complex mediates photoperiodic growth by regulating *LAP1*. PtFD2 (FDL1) participates in the control of adaptive response and bud maturation pathways via interaction with ABI3. **(C)** EjFD interacts with EjTFL1s or EjFT1 to suppress the expression of *EjAP1-1*, which leads to the inhibition of loquat flower bud differentiation. EjFD-EjFT2 promotes floral bud formation by promoting the expression of *EjAP1–1* and *EjAP1-2*, which is regulated by photoperiod and GA signals.

In pea, *VEGETATIVE2* (*VEG2*), which is homologous to *FD*, plays a central role in regulating meristem identity throughout the development of the compound inflorescence. VEG2 interacts with FTb2 in the shoot apex to promote primary (I_1_) inflorescence meristem identity through *DETERMINATE* (*DET*), *LATE FLOWERING* (*LF*), *FTa1*, and *FTc*. VEG2 interacts with FTa1 at the shoot apex to promote secondary (I_2_) inflorescence meristem identity via *VEG1* and *FTc*. *veg2* mutant transforms I_2_ into I_1_ inflorescence meristems (Sussmilch et al., 2015). VEG2 is also involved in the regulation of floral architecture through the regulation of MADS-box genes such as *PIM* (*AP1*), *AP3*, and *SEP1. veg2* mutant has defective sepals and petals, fused floral organs, reduced organ numbers, and malformed organs (Sussmilch et al., 2015) (**Figure 3A**).

*MtFDa*, an ortholog of pea *VEG2/PsFDa*, plays a key role in inflorescence development in *Medicago truncatula*. *mtfda* has tertiary branches and bracts that transform into compound leaves, suggesting that *MtFDa* is required for I_2_ inflorescence meristem identity and development. *mtfda* and *mtfta1* flower later than WT, the double mutant *mtfda/mtfta1* never forms flowers, and no floral transition in *mtfda/mtfta1* happens (Cheng et al., 2021). The phenotype of *mtfda/mttfl1* double mutant phenotype is similar to that of *mtfda*. The I_1_ inflorescence shows indeterminate growth, indicating that *MtFDa* is epistatic to *MtTFL1* for I_1_ indeterminacy. *MtFDa* and *MtFULc* co-determine I_2_ identity. The I_2_ inflorescence of the *mtfda/mtfulc* double mutant transforms into an I_1_-like vegetative structure, producing compound inflorescences, compound leaves, and indeterminate apices. The *mtfda/mtfulc/mttfl1* triple mutant has a similar flowering time, inflorescence, and flower as *mtfda*, indicating that *MtFDa* has an epistatic effect on *MtFULc* (Cheng et al., 2018). Collectively, *MtFDa* plays a key role in inflorescence development, functions in coordination with *MtFULc* for I_2_ inflorescence meristem identification, and is epistatic to *MtTFL1* for I_1_ indeterminacy (Cheng et al., 2018, 2021).

*PaFDL1* and *PaFDL2* are *FD* homologs of the London plane that participate in flower organ development. Overexpression of *PaFDL1* and *PaFDL2* in tobacco leads to extended stigmas, and curled petals at the tips (Cai et al., 2021).

### 5.2 Inflorescence structure and plant height

FD-like proteins participate in inflorescence development, stem growth, and seed development. The inflorescences of *Nicotiana tabacum NtFD1*, *NtFD3*, and *NtFD4* overexpression lines are condensed and the pedicles, peduncles, and internodes are short, resulting in a bushy, bunch-like architecture. They flower earlier than the WT, fewer leaves are produced before flowering, and differentiation of axillary meristems is also premature compared with WT (Beinecke et al., 2018).

Rice *OsFD7* RNAi lines have longer and denser panicles, more florets, elevated seed size and weight, and more seeds. The transcription levels of *OsMADSs* are down-regulated in *OsFD7* RNAi lines (Kaur et al., 2021). *OsFD2* inhibits the developmental shift from inflorescence branch meristem to floral (or spikelet) meristem in panicle branches, which leads to plentiful spikelets or secondary branches and a dense panicle phenotype with smaller leaves (Tsuji et al., 2013). The overexpression of bamboo *BtFD1* in *Arabidopsis* also leads to dwarfism and an apparent reduction in the length of the flowering stalk and number of flowers per plant (Dutta et al., 2021). The overexpression of *FD* by the 35S promoter in *Arabidopsis* results in dwarf plants (Abe et al., 2005). Overexpression of *FD* and *FDP* (*At2g17770*/*AtbZIP27*) in rice causes a reduction in plant height and spikelet size with decreased expression of genes involved in cell elongation without significant flowering time alteration, which is linked to impaired gibberellin biosynthesis in plants (Jang et al., 2017). Romera-Branchat et al. (2020) reported that FD and FDP bind to genes involved in water deprivation and hormonal pathways, including gibberellic acid, ABA, and jasmonic acid. These results provide evidence of crosstalk between the regulation of plant morphogenesis and hormone signaling pathways.

In soybean, overexpression of *GmFDc1* leads to fewer nodes (Yue et al., 2021). GmFT5a interferes with the binding of Dt1 to GmFDc1 and enhances the positive effect of GmFDc1 on *GmAP1* expression (Yue et al., 2021) (**Figure 2E**). *Dt1* controls stem growth habit and flowering time and strongly influences soybean grain yield (Liu et al., 2010; Yue et al., 2021). Mutations in the recessive alleles of *gmft2a* and *gmft5a* delay flowering and increase node number, branch number, and yield (Li et al., 2021). Thus, *GmFDc1* appears to contribute significantly to soybean plant architecture and yield.

### 5.3 Photoperiod signal and plant growth

In poplar, overexpression of *PtFD1* (*FDL2*) results in severe dwarfing under a LD photoperiod, however, SD-induced growth arrest and bud formation are lost in *PtFD1* (*FDL2*)-overexpressing lines (Parmentier-Line and Coleman, 2015; Tylewicz et al., 2015). *PtFD2* (*FDL1*) overexpression resulted in a delayed SD response compared to WT. FT- PtFD2 (FDL1) complex mediates photoperiodic growth by regulating *Like AP1* (*LAP1*). PtFD2 (FDL1) also participates in controlling adaptive responses and bud maturation pathways by interacting with ABSCISIC ACID INSENSITIVE 3 (ABI3), a component of ABA signaling (Tylewicz et al., 2015) (**Figure 3B**). Loquat EjFD suppresses *EjAP1–1* expression by interacting with EjTFL1s or EjFT1, inhibiting loquat flower bud differentiation. Conversely, EjFD-EjFT2 promotes floral bud formation under the regulation of photoperiod and GA signals (Jiang et al., 2020, 2024) (**Figure 3C**).

## 6 Competition of FT and TFL1 for FD binding

### 6.1 The balance between vegetative and reproductive stage

In contrast to FT and TSF, TERMINAL FLOWER 1 (TFL1) and BROTHER of FT and TFL1 (BFT) are floral repressors in the PEBP family. These PEBPs have conserved 14-3–3 binding motifs and interact with FD (Yoo et al., 2010; Hanano and Goto, 2011; Ryu et al., 2014). TFL1 competes with FT for FD binding and represses the transcription of floral meristem identity genes such as *LFY* and *AP1* (Hanano and Goto, 2011) (**Figure 2A**). TFL1 interacts with unphosphorylated FD via 14-3–3 proteins, suggesting that the inactive FD/14-3-3/TFL1 ternary complex may be present in the basal state of the SAM. Only when FD is phosphorylated can FT form an active complex with the 14-3–3 proteins to induce flowering. The efficient phosphorylation of T282 in FD is calcium-dependent. This requirement may help prevent the premature induction of flowering (Kawamoto et al., 2015). Under high salinity conditions, BFT delays flowering. The relative transcript levels of BFT are higher than those of FT and the high-level BFT protein competes with FT for FD binding in the SAM (Ryu et al., 2014) (**Figure 2A**). Similar to FD, FDP is phosphorylated by CPK33, forming a complex with FT and TFL1 in a phosphorylation-dependent manner. The weak late-flowering phenotype of *cpk33–1* may be due to the combined effect of the florigen and anti-florigen complex formation of FD and FDP (Kawamoto et al., 2015).

The rice TFL1-like protein RICE CENTRORADIALIS (RCN) competes with Hd3a for 14-3–3 binding. RCN protein transports from the vasculature to SAM to form the “florigen repression complex” (FRC) with 14-3–3 and OsFD1 and then represses florigenic activity. The balance between FRC and FAC depends on the ratio of Hd3a to RCN and regulates the development of SAM. In the vegetative phase, FRC are formed. Upon reaching the SAM, Hd3a competes with RCN for FAC formation. When the balance shifts to FAC, the reproductive program begins (Kaneko-Suzuki et al., 2018) (**Figure 2B**).In soybean, Dt1 and GmFT5a have opposite functions. Dt1 complementary lines produce more nodes and flower later than WT (Yue et al., 2021). *gmft5a* delays flowering and increases node number (Li et al., 2021). Dt1 interacts with GmFDc1 and binds to ACGT cis-elements in the promoter of *GmAP1a* to repress its activity. GmFT5a interferes with the binding of Dt1 to GmFDc1 and enhances the positive effect of GmFDc1 on *GmAP1* expression (Chen et al., 2020b; Yue et al., 2021). TFL1c and TFL1d, homologs of Dt1, interact with GmFDc1 and binds to ACGT cis-elements in the promoter of *GmAP1a* to repress its activity (Wang et al., 2023) (**Figure 2E**). TFL1c and TFL1d might also compete with GmFT5a for GmFDc1 binding. EjTFL1s inhibit loquat flower bud differentiation through EjFD binding and suppression of *EjAP1-1* (Jiang et al., 2020). In contrast, EjFT1 and EjFT2 interact with EjFD but have opposing effects: EjFT2-EjFD activates *EjAP1–1* and *EjAP1-2*, while EjFT1-EjFD represses *EjAP1-1*. EjFT1 may resemble EjTFL1s in promoting vegetative growth. The competitive interaction between EjFT1 and EjFT2 with EjFD regulates floral bud differentiation, with EjFT2 promoting flowering and EjFT1 supporting vegetative growth (Jiang et al., 2024) (**Figure 3C**). Protein structural analysis of EjFT1 and EjFT2 suggests that differences in amino acid residues at Val123/Leu123, Ser157/Ala157, and Val158/Ala158 may be the reason for their functional differences (Jiang et al., 2024).

### 6.2 Environment cues and the antagonistic regulation

In *Brachypodium distachyon*, FTL9 and FD1 form an FAC that induces *VRN1* and *FUL2* expression, promoting flowering under SD conditions. Under LD conditions, however, *FTL9* inhibits flowering. The FTL9-FD1 complex is less potent than the FT1-FD1 complex in inducing flowering. Overexpression of *FTL9* disrupts FT1-FD1 complex formation by competing for FD1 binding, leading to reduced *VRN1* expression and delayed flowering (Qin et al., 2019).

Kiwifruit CEN and FT interact with AcFD but exhibit distinct temporal expression patterns. *FT* is specifically induced in dormant buds during winter chilling, promoting dormancy release, whereas *CEN* transcripts accumulate in latent buds during summer but decrease in autumn prior to dormancy establishment. These contrasting expression patterns and functional roles suggest that CEN and FT may act as antagonistic regulators in kiwifruit development (Varkonyi-Gasic et al., 2013).

## 7 FD directly regulates genes related to flowering and endogenous signalling

The direct target genes of TFL1-FD and FT-FD complexes have been detected by ChIP-seq and RNA-seq experiments. The results reveal that the target genes have a prominent role in cell signaling, including flowering time genes (*PRR7*, *CONSTANS* (*CO*), and *GIGANTEA* (*GI*)) and floral identity genes (*LFY*, *AP1*, *FUL*, and *LATE MERISTEM IDENTITY 2* (*LMI2*)). TFL1-FD represses while FT-FD activates the target genes (Collani et al., 2019; Goretti et al., 2020; Romera-Branchat et al., 2020; Zhu et al., 2021). CO and GI are regulators of flowering by activating *FT* in the photoperiodic pathway. CO and GI might act in feedback regulatory pathways with FT-FD complexes. The *PRR7* gene is involved in the regulation of the plant biological clock and affects the circadian rhythmicity (Li et al., 2016). Current studies have not directly revealed the regulatory relationship between PRR7 and FT-FD. *LFY*, *AP1*, *FUL*, and *LMI2* who are involving in the regulation of plant structure, floral meristem differentiation, and flowering time, have been proved to be the direct targets of TFL1-FD and FT-FD complexes in many plant species (Abe et al., 2005; Wigge et al., 2005; Ahn et al., 2006; Li and Dubcovsky, 2008; Taoka et al., 2011; Sussmilch et al., 2015; Collani et al., 2019; Takeshima et al., 2019; Yue et al., 2021; Dutta et al., 2021; Ye et al., 2023; Park et al., 2023).

TFL1-FD and FT-FD complexes also bind to genes linked to phytohormone biosynthesis, signaling, and response (auxin, ABA, brassinosteroid, cytokinin, jasmonic acid, and strigolactone) as well as genes linked to sugar signaling (Trehalose-6-phosphatases (*TPP*) genes) (Collani et al., 2019; Goretti et al., 2020; Zhu et al., 2021). The plant height, branch growth, bud growth, spikelet size, and tolerance to drought and salt stress influenced by *FD* gene family are reported to be related to these endogenous signals (Tylewicz et al., 2015; Jang et al., 2017; Li et al., 2017; Jiang et al., 2020, 2024).

## 8 Conclusions and perspectives

Flowering time of crops is essential for adaptation and yield. Early flowering enhances the efficiency of reproductive development, whereas delayed flowering facilitates the accumulation of materials through prolonged vegetative growth (Wang et al., 2024). Many genes regulating flowering time in crop species have been utilized in molecular breeding, such as *Heading date 1* (*Hd1*) and *Grain number, plant height, and heading date 7* (*Ghd7*) in rice (Yano et al., 2000; Xue et al., 2008), *Vernalization 1* (*VRN1*) and *Photoperiod-D1* (*Ppd-D1*) in wheat (Yan et al., 2003; Beales et al., 2007), *J* and *SOC1* in soybean (Lu et al., 2017; Kou et al., 2022). However, *FD* genes act as a floral activator are seldom used. Perhaps *FD*’s function is less studied than *FT*. In the future, diverse *FD* alleles in crop germplasm resources should be utilized in modern breeding.

The bZIP transcription factor *FD* is a central regulator of yield traits, such as plant height, inflorescence structure, and seed development. In barley, bZIP transcription factors have been implicated in pre-anthesis tip degeneration (PTD), a programmed process critical for seed number and yield. However, the precise molecular functions and regulatory pathways remain unclear (Shanmugaraj et al., 2023). Interestingly, unlike in other species where *TFL1* homologs control inflorescence determinacy, AP2L-5 (an AP2-family transcription factor) serves as the primary regulator of determinate/indeterminate inflorescence fate in barley (Zhong et al., 2021). This suggests that FD-like proteins may operate through distinct regulatory mechanisms in barley compared to other crops.

*FD* is essential for vegetative growth, overexpressing *FD* in *Arabidopsis*, rice, tobacco, bamboo, or poplar lead to dwarf phenotype. During the “Green revolution”, the utilization of “dwarfing genes” facilitated the breeding of novel cultivars in rice, wheat, and maize with enhanced resistance to lodging. The implementation of optimal plant densities contributed to a substantial augmentation in crop productivity (Hou et al., 2024). Therefore, *FD* might be a promising “dwarfing gene” in crop breeding and production increasing.

*FD* can respond to environmental factors such as photoperiod, temperature, phytohormones, and abiotic stresses (Zhu et al., 2021). This phenomenon suggests that FD may serve as a valuable genetic resource for enhancing plant adaptability to adverse environmental conditions. Whereas, *FD*’s potential has not been fully explored. It has been reported that other Group A members of bZIP transcription family, such as ABF1/AtbZIP35, ABF2/AREB1/AtbZIP36, ABF3/AtbZIP37, ABF4/AREB2/AtbZIP38, and ABI5/DPBF1/AtbZIP39 are associated with ABA and stress signaling or ABA-dependent seed maturation and germination (Choi et al., 2000; Uno et al., 2000; Skubacz et al., 2016). The functions and molecular mechanisms of *FD* homologs in relevant pathways might be a promising direction for future research.

Overall, a comprehensive overview of *FD* gene family will not only deepen our knowledge of the diverse roles executed by *FD* gene family in flowering, plant development, and environment signaling responses but will also facilitate the exploration of innovative strategies to improve crop productivity in challenging environments.

## Supplementary material

Supplementary Table 1 List of genes in **Figure 1**.
